# Dual Field-of-View Off-Axis Spatially Multiplexed Digital Holography Using Fresnel’s Bi-Mirror

**DOI:** 10.3390/s24030731

**Published:** 2024-01-23

**Authors:** Lavlesh Pensia, Manoj Kumar, Raj Kumar

**Affiliations:** 1 CSIR—Central Scientific Instruments Organisation, Sector 30C, Chandigarh 160030, India; lavleshpensia25@gmail.com; 2 Academy of Scientific and Innovative Research (AcSIR), Ghaziabad 201002, India; 3 Department of Systems Science, Graduate School of System Informatics, Kobe University, Kobe 657-8501, Japan; 4 Center of Optical Scattering Image Science, Kobe University, Kobe 657-8501, Japan

**Keywords:** digital holography, multiplexed holography, Fresnel bi-mirror, Fresnel diffraction method

## Abstract

Digital holography (DH) is an important method for three-dimensional (3D) imaging since it allows for the recording and reconstruction of an object’s amplitude and phase information. However, the field of view (FOV) of a DH system is typically restricted by the finite size of the pixel pitch of the digital image sensor. We proposed a new configuration of the DH system based on Fresnel’s bi-mirror to achieve doubling the camera FOV of the existing off-axis DH system which leveraged single-shot acquisition and a common-path optical framework. The dual FOV was obtained by spatial frequency multiplexing corresponding to two different information-carrying beams from an object. Experimental evidence of the proposed dual FOV-DH system’s viability was provided by imaging two different areas of the test object and an application to surface profilometry by measuring the step height of the resolution chart which showed excellent agreement with an optical profiler. Due to the simple configuration, the proposed system could find a wide range of applications, including in microscopy and optical metrology.

## 1. Introduction

Digital holography (DH) is a versatile, 3D imaging technique that can precisely measure the amplitude and phase of light by utilizing the interference phenomena [1,2,3,4,5,6]. Beyond conventional systems, access to complex amplitude in digital form has a wide range of applications. For example, it allows for numerical propagation of the wavefront over the measurement area, which enables 3D shape recognition [7], depth of field extension [8,9], and biological cell imaging [10], to name a few. However, despite the significant benefits of DH, the performance and complicated structure of the interferometric setup are considered to be the primary factors impeding the system’s use in industry. With the recent developments in technology, such as the small pixel size of the image sensor and the high coherence of compact light sources, DH systems can be implemented with only a few key components. The field of view (FOV) of the DH system is still limited due to the finite pixel pitch of the image sensor. As a result, presently DH applies to some limited applications only. Non-destructive testing (NDT) and optical metrology of various manufactured products are of great importance. Over the past two decades, several methods have been proposed to expand the FOV of DH systems. These methods are mainly categorized into three approaches: multiplexing methods [11,12,13,14,15,16,17,18,19,20,21], lens-based methods [22,23,24], and image-stitching methods [25]. In multiplexing methods such as spatial [11,12,13,14,15], angle or frequency [16,17,18,19,20], and wavelength division [21], different areas of the object (FOVs) are strategically folded in such a way that they overlap with each other at the image sensor’s FOV. Several optical configurations based on diffraction grating [18,19], retro-reflector [12], synthetic aperture technique [26], lenses [22], multifunctional holographic optical elements [23], etc., have been reported to extend the FOV of DH systems. However, most of the reported setups are either complicated or bulky and demand stringent requirements in their realization. For example, in the grating-based method [18], the grating must be placed near the object, which is not always possible for all objects. If the grating is not correctly aligned in contact with the object surface, the cross-talk problem appears in the reconstruction. By employing beam folding mirrors, Tayebi et al. [16,17] extended the FOV of DH to double and triple the sensor FOV by recording two [16] and three [17] spatially multiplexed holograms, respectively. The optical arrangement requires a tube lens, a pinhole (for generating the reference beam), and a 4-*f* optical system [16], which makes the system bulkier and more complicated. Lens-based systems [22,23,24] reported for extending the FOV require a special lens or lens system and stringent parameters including focal length, position, and diameters, etc., [22] which introduces several aberrations, and the overall system configuration is still bulkier. The image stitching methods [25] involve moving the position of either the object under investigation or the image sensor, to record a series of holograms, and finally stitch the reconstructed images. This approach necessitates expensive mechanical equipment and time-consuming computations, and therefore, is not suitable for investigations of fast transient phenomena. Several other innovative techniques such as post-processing computational methods [27,28,29,30,31,32,33,34] have been proposed to improve the resolution and FOV of DH systems.

In this work, we present a novel method for spatially multiplexing two FOVs into a single off-axis hologram using a Fresnel’s bi-mirror to extend the FOV of DH by doubling the sensor’s FOV. The proposed configuration not only reduced the number of components used in the setup but also increased the FOV of the setup in a compact and efficient manner. The proposed system showed greater stability due to the common-path optical layout, compactness, and most significantly, dual FOV capabilities. We experimentally validated the proposed method to dual the FOV of the DH system by imaging different areas of phase objects including a resolution chart. Furthermore, an application to measure the surface topography was experimentally demonstrated by the dual FOV-DH system.

## 2. Materials and Methods

The digital holograms were generated by coherent mixing of the reference beam *E_r_*(*x*, *y*) and object beam *E_o_*(*x*, *y*), and recorded by an image sensor. The object and reference beams could be represented as [20]:

(1) $$E_{o}(x,y)=A_{o}(x,y)\exp(-j\phi_{o}(x,y))$$

(2) $$E_{r}(x,y)=A_{r}(x,y)\exp(-j\phi_{r}(x,y))$$

where *A_r_*(*x*, *y*) and *ϕ_r_*(*x*, *y*) represent the amplitude and phase distributions of the reference beam, and *A_o_*(*x*, *y*) and *ϕ_o_*(*x*, *y*) represent the amplitude and phase distributions of the object beam, respectively, and *j* = √−1. The intensity distribution of interference patterns *E_h_*(*x*, *y*) were obtained at the hologram plane after coherent mixing of the object and reference beams, which could be represented as [20],

(3) $$E_{h}(x,y)=\left\{E_{o}(x,y)+E_{r}(x,y)\right\}{\left\{E_{o}(x,y)+E_{r}(x,y)\right\}}^{*}$$

(4) $$E_{h}(x,y)={\left|E_{o}(x,y)\right|}^{2}+{\left|E_{r}(x,y)\right|}^{2}+E_{o}(x,y)E_{r}^{*}(x,y)+E_{o}^{*}(x,y)E_{r}(x,y)$$

In Equation (4), the first two terms ${\left|E_{o}(x,y)\right|}^{2}+{\left|E_{r}(x,y)\right|}^{2}$ on the right-hand side are the constant terms of intensity, $E_{o}(x,y)E_{r}^{*}(x,y)$ and $E_{o}^{*}(x,y)E_{r}(x,y)$ represent the complex amplitude of the object beam and its complex conjugate, respectively. This intensity distribution was digitized by the image sensor and the recorded digital hologram was stored in a computer [20].

In the DH, the recorded digital holograms can be reconstructed using various numerical reconstruction methods such as the angular spectrum method (ASM), Fresnel diffraction method (FDM), and convolution method. In the proposed system, the FDM [35] was used for amplitude and phase reconstruction, because it satisfies the distance criterion for propagation. Contrarily, the ASM and convolution-based methods are exact methods that are constrained to shorter propagation distances and induce aliasing in reconstruction for larger objects over longer distances, but they significantly increase the execution time in numerical reconstruction processing. As a result, FDM was considered the most appropriate for this work. The complex amplitude of the object recorded in digital holograms could be reconstructed using the FDM, represented as

(5) $$O_{o}\left(\xi ,\eta \right)=\frac{\exp\left(-jkz\right)}{j\lambda z}\exp\left(\frac{-j\pi \left(x^{2}+y^{2}\right)}{\lambda z}\right)\times FT\left[E_{h}\left(x,y\right)\exp\left(\frac{-j\pi \left(x^{2}+y^{2}\right)}{\lambda z}\right)\right]$$

where *O_o_*(*ξ*, *η*) and *E_h_*(*x*, *y*) are complex amplitude distributions at the object plane and hologram plane, respectively. *FT* represents the Fourier transform, *k* = 2*π*/*λ*, *λ* is the source wavelength, and *z* is the propagation distance [20]. For reconstruction, conjugate orders were filtered out in the Fourier domain and were propagated using the FDM. This filtering removed the constant DC terms and other conjugate orders.

**Figure 1:**
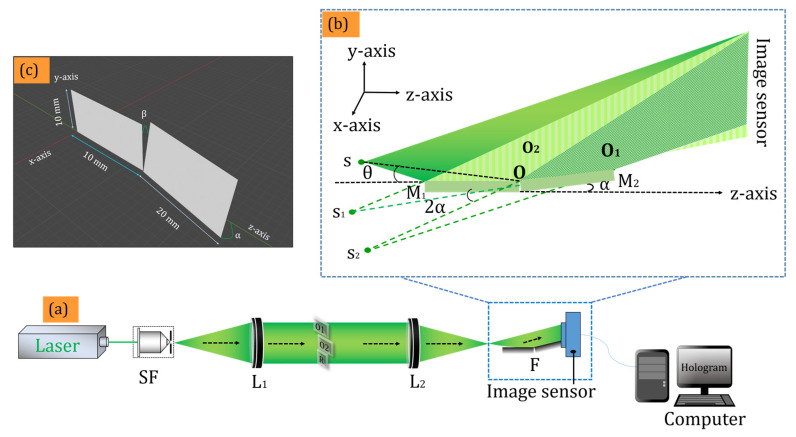
(**a**) Schematic of the proposed Fresnel’s bi-mirror-based off-axis DH experimental set-up; (**b**) zoomed area of the two FOV generations by Fresnel bi-mirror; (**c**) schematic arrangement of the Fresnel bi-mirror.

Figure 1 depicts the proposed experimental setup for recording a multiplexed digital hologram.

**Figure 2:**
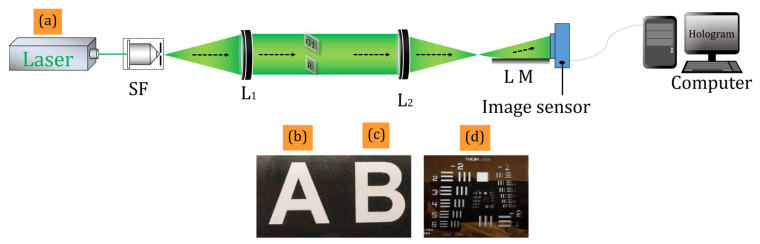
(**a**) Lloyd mirror interferometer-based digital holographic setup; (**b**–**d**) the objects used in the experiments. SF-spatial filter, L-Lens, F-Fresnel’s bi-mirror, LM-Lloyd mirror.

A solid-state laser (LCX-532S, OXXIUS, Lannion, France) with a wavelength of 532 nm was used as the light source. The laser beam was expanded by a spatial filter with a 40× microscopic objective and a 5 µm pinhole. The expanded beam was collimated by a lens (*L*_1_) of focal length 200 mm. The collimated beam had a diameter of ~50 mm, whereas the active area of the image sensor was 5.4 mm × 4.5 mm (sensor FOV). Therefore, the image sensor recorded only a small portion of the optical field, i.e., equivalent to the sensor FOV. We compared the experimental findings with a similar DH system, the Lloyd mirror interferometer-based DH system. Figure 2a depicts the Lloyd mirror interferometer-based digital holographic setup for recording a digital hologram.

In order to record a larger optical FOV, we use the optical multiplexing method in which two different areas of the optical FOV were projected onto the sensor FOV by using a Fresnel bi-mirror. At the same time, a small portion of the object beam, free from object information that served as a reference beam, was also projected on the sensor FOV. Therefore, the optical layout of the system was common-path and hence it was expected to provide greater stability as compared to the two beam DH systems. Alternatively, the collimated beam was virtually divided into three beams: the upper two beams functioned as the object beams (*O*_1_ and *O*_2_), and the third beam functioned as a reference beam (R), as schematically shown in Figure 1a. Two English letters, A and B, each of size ~5.2 mm × 4.1 mm, were placed in the object beams. These letters were printed on a thin transparent sheet with a refractive index of ~1.50. The second lens (*L*_2_) of focal length 200 mm converged this collimated beam. The key component of the proposed setup, which was a Fresnel bi-mirror, was placed at a distance of 2 mm from the Fourier plane of the lens *L*_2_. This distance may have varied according to the parameters (focal length and aperture) of *L*_2_. The diverging beam after *L*_2_ comprised *O*_1_, *O*_2_, and R, reflected differently by the Fresnel bi-mirror, which was composed of mirrors *M*_1_ and *M*_2_, as schematically shown in Figure 1b. Upon reflection, the beams appeared to originate from virtual point sources, namely *S*_1_ and *S*_2_. Multiple interference patterns were generated on the hologram plane due to the overlap between the beams from the point source s and the virtual sources *S*_1_ and *S*_2_. The dimensions of the two mirrors were 10 mm × 10 mm and 10 mm × 20 mm, respectively, as shown in Figure 1c. The image sensor (CMOS sensor, resolution 2592 × 1944, pixel pitch 2.2 µm) was positioned at a distance of ~10 mm from the Fresnel bi-mirror, where the interaction of the two FOVs was created by spatial multiplexing of the two holograms. The reference beam was allowed to interfere, with the help of Fresnel’s bi-mirror, in common-path and off-axis DH geometry with the two object beams (*O*_1_ and *O*_2_), at the active region of the image sensor to form a multiplexed hologram. The fringe widths of the interference between light from the virtual sources *S* and *S*_1_; *S*_1_ and *S*_2_, and *S* and *S*_2_, respectively, could be determined by the equations:

(6) $$\beta_{\left(SS_{1}\right)}^{\prime }=\frac{\lambda D}{2r\theta }$$

(7) $${\beta^{\prime \prime }}_{\left(S_{1}S_{2}\right)}=\frac{\lambda D}{2r\sqrt{\alpha^{2}+\beta^{2}}}$$

(8) $${\beta^{\prime \prime \prime }}_{\left(S_{1}S_{2}\right)}=\frac{\lambda D}{2r\sqrt{\theta^{2}+\alpha^{2}+\beta^{2}}}$$

where *D* represents the distance between two-point sources, *λ* is the wavelength of the light source, *θ* is the angle between the *z*-axis and the virtual source *s*; *α* and *β* are the angles that mirror M_2_ makes with the *z*-axis and the *y*-axis, respectively; *r* is the distance from the point *O* to virtual sources (*S*_1_, *S*_2_, and *S*_3_). 

The maximum angle between the object and reference beams must not exceed the image sensor’s resolution limit *ϕ* = sin^−1^(*λ*/2Δ*x*), where Δ*x* is the pixel pitch of the image sensor. The angles between various beams at the sensor plan were:

(9) $$\theta_{\left(SS_{1}\right)}=\sin^{-1}\left(\frac{2r\theta }{D}\right)$$

(10) $$\theta_{\left(S_{1}S_{2}\right)}=\sin^{-1}\left(\frac{2r\sqrt{\alpha^{2}+\beta^{2}}}{D}\right)$$

(11) $$\theta_{\left(SS_{2}\right)}=\sin^{-1}\left(\frac{2r\sqrt{\theta^{2}+\alpha^{2}+\beta^{2}}}{D}\right)$$

where *θ*(*SS*_1_), *θ*(*S*_1_*S*_2_), and *θ*(*SS*_2_) are angles between the interfering beams (*S* and *S*_1_), (*S*_1_ and *S*_2_), and (*S* and *S*_2_), respectively. 

The maximum angle between the object and reference beam must not exceed 6.9°, a limit imposed by the pixel pitch of the image sensor (2.2 µm) and wavelength (532 nm) of the laser in order to reconstruct the faithful image from the recorded digital hologram. In the proposed setup, one mirror (*M*_1_) was aligned parallel to the system’s optical axis, while the second mirror (*M*_2_) made a *α* = 3° angle with the *z*-axis and *β* = 1° angle with the *y*-axis; however, other combinations could also be explored. Thus, the collimated beam was divided into three beams that interfered at the image sensor plane, to generate an off-axis multiplexed hologram. Theoretical values of the angles between the interfering beams at the hologram plane were obtained using Equations (6)–(8). After passing through L_2_, the reference beam went directly to the active region of the image sensor without being reflected by the Fresnel’s bi-mirror. The right portion (*O*_1_) and central portion (*O*_2_) of the collimated beam were reflected by the Fresnel’s bi-mirror towards the image sensor and formed an off-axis multiplexed digital hologram by interfering with the reference beam at the hologram plane. 

The space bandwidth product (SBP) is a product of the active area (as a function of the number of pixels) of the sensor and it’s spatial frequency bandwidth, in the off-axis multiplexing hologram. The bandwidth utilization was defined as the area ratio of the conjugate terms to the camera’s bandwidth (*Bc*) [15,36,37]. The pixel size of the image sensor was Δ*x*, and the number of pixels along the *x*-axis and *y*-axis was *M* × *N*, respectively. In the diffraction-limited optical system, the distribution of the spectrum was a circle; its area could be calculated as $\pi {\left(B_{o}/2\right)}^{2}$ where *B_o_* is the bandwidth of the object beam. The bandwidth utilization of the off-axis hologram was $2\pi \Delta x^{2}{\left(B_{o}/2\right)}^{2}$. Its SBP could be calculated as $\pi {\left(B_{o}/2\right)}^{2}MN\Delta x^{2}$. In the spatial frequency domain, the digital hologram occupied an area with *M* × *N* pixels. The horizontal and vertical bandwidths *Bo* of each conjugate term were *M*/4 and *N*/4, respectively. The shape of the conjugate term was ellipse due to the number of pixels M along the *x*-axis, which was different from the number of pixels *N* along the *y*-axis. Its area was $\pi \left(M/8\right)\left(N/8\right)$. The bandwidth utilization of two multiplexed holograms, in this work, was 4πMN/64MN=19.63%.

The quality of the reconstructed image may have been degraded by the cross terms, but the experimental setup was designed and developed in such a way that there was a sufficiently large angle between the beams *O*_1_ and *O*_2_, which created a separation in their Fourier spectra. As a result, the cross-interference orders did not affect the quality of the reconstructed images. In comparison to the works reported in the literature [16,17], the proposed system was more compact and utilized a simple Fresnel bi-mirror instead of several tilted mirrors. Secondly, both object beams (*O*_1_ and *O*_2_) superimposed with a minimal angle of approximately 1°. As a result of this small angle, the spatial frequency of their interference fringes converged near the DC frequency in the spatial spectrum. The developed holographic system was utilized for imaging as well as non-destructive testing applications. The latter case relies on the principle of digital holographic interferometry (DHI), in which two digital holograms are recorded corresponding to the two states of an object, i.e., without external loading of the object and with loading the object. 

## 3. Results and Discussion

### 3.1. Field of View

**Figure 3:**
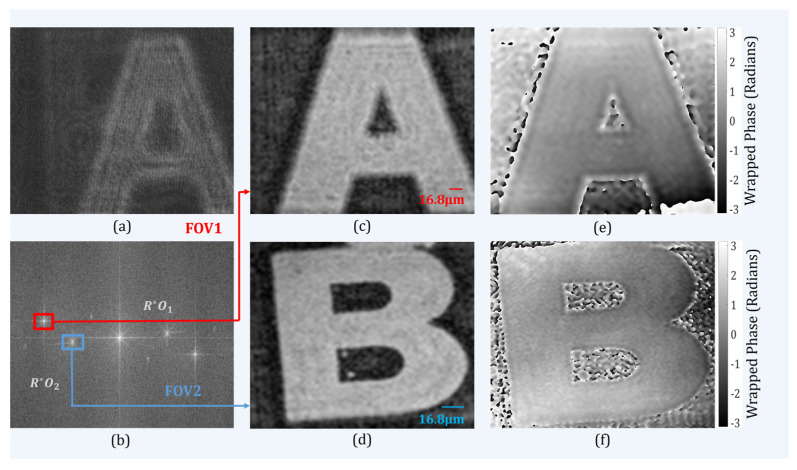
Experimental results obtained with the proposed setup. (**a**) Recorded digital hologram; (**b**) Fourier spectrum of (**a**); (**c**,**d**) Numerically reconstructed amplitude images corresponding to two FOVs; and (**e**,**f**) wrapped phase image for two FOVs.

Figure 3a shows a recorded multiplexed digital hologram in which it is clearly seen that the information of multiple objects was superimposed onto the same active region of the image sensor (sensor FOV). As the reference beam was common for both object beams, the image sensor recorded three off-axis interference patterns. The three sets of fringes could be directly visualized in the spatial frequency domain of the multiplexed hologram. The reference beam (R) and two object beams (*O*_1_ and *O*_2_) interfered with each other to form two interference patterns; a third interference pattern was formed due to the superposition of the two object beams with each other. The recording and reconstruction distances used in this work were 280 mm. Figure 3c,d depict the amplitude distribution of the reconstructed images corresponding to the two FOVs, which was equivalent to doubling the recording area when compared to the conventional holographic setup. Figure 3e,f show the retrieved wrapped phase images corresponding to the two FOVs. 

**Figure 4:**
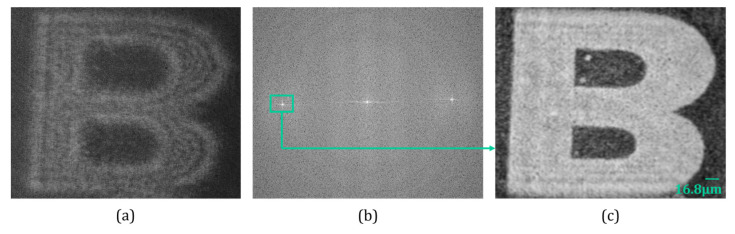
Experimental results obtained with the Lloyd mirror interferometer setup: (**a**) recorded digital hologram, (**b**) Fourier spectrum of recorded digital hologram (**a**,**c**) reconstructed amplitude image.

Here, we considered the Lloyd mirror interferometer [38,39,40] (see schematic experimental setup in Figure 2a) for comparison as its optical configuration is quite similar to Fresnel’s bi-mirror configuration [41,42]. In the case of the Lloyd mirror interferometer-based holographic setup [39,40] one of the beams was reflected by the Lloyd mirror where only a small part of the object corresponding to the active area of the image sensor could be recorded successfully. Figure 4a shows the recorded hologram with a Lloyd mirror interferometer-based holographic setup. The Fourier spectrum of the recorded hologram is depicted in Figure 4b, where ±1 orders are clearly seen. One conjugate order was filtered out from the Fourier domain and subsequently numerically reconstructed using the FDM, as shown in Figure 4c.

Thus, the FOV of the Lloyd mirror interferometer-based holographic optical system is one-half (or equivalent to the sensor FOV) of the proposed system wherein Fresnel’s bi-mirror directed two object beams onto the same image sensor. Since the sensor’s FOV was limited due to the constrained specifications (finite pixel size of the image sensor and size of the active region), the proposed off-axis DH system had the ability to record and retrieve the dual optical FOV. Hence, the system showed a capability of dual FOV by using spatial multiplexing property. 

### 3.2. Resolution

**Figure 5:**
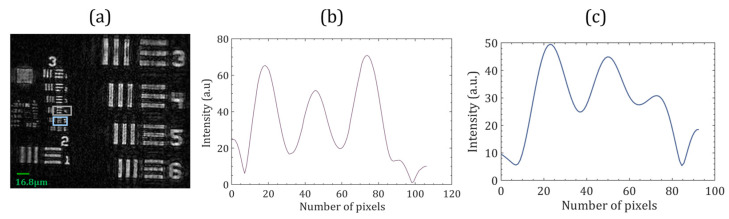
(**a**) Experimental results of the 1951 USAF resolution test targets. (**b**,**c**) show the intensity profiles along the elements 4 and 5, respectively, of group 3.

A USAF resolution chart (Model number: R3L3S1N, Thorlabs Inc., Newton, NJ, USA) was used as a test object to demonstrate the resolution of the proposed system. Figure 5a shows the numerically reconstructed image of the test target resolution chart. The size of the USAF resolution chart was larger than the size of the object beam, so to test the optical system’s resolution, the right and central parts of the collimated beam were used as object beams, and the left part served as a reference beam for this experiment. The smallest resolvable group 3, element 4, corresponded to a resolution of 11.30 lines per mm (44.6 µm), as shown in Figure 5b. The proposed system could not resolve the line profile of group 3, element 5, shown in Figure 5c. The Abbe diffraction limit is λ/*NA* for coherent illumination, where *NA* represents numerical aperture. At a wavelength of 532 nm, the acceptable resolution limit of the proposed system was 44.6 µm (with *NA* of 0.13). 

Therefore, it was observed that the smallest resolution (40 µm) image by the theoretical diffraction limit could be provided by the proposed system. Two digital holograms multiplexed into a single hologram affected the resolution of the reconstructed image.

### 3.3. Temporal Stability

**Figure 6:**
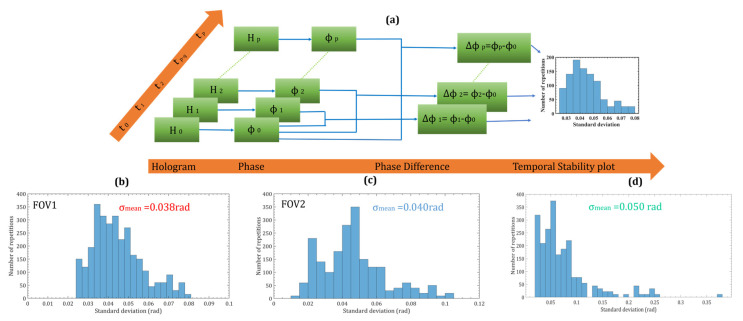
(**a**) Flowchart of the methodology used to study the temporal stability of the proposed optical system. Digital holograms recorded by using off-axis digital holography at different instants of time were assigned frame numbers from 0 to p. (**b**,**c**) temporal stability histogram of FOV1 and FOV2 show the proposed setup, (**d**) temporal stability histogram of the Lloyd mirror interferometer-based holographic optical system.

The temporal stability of the common-path dual FOV-DH system was measured by recording a series of holograms and calculating the phase difference distributions. A time series of digital holograms (H_0_, H_1_, H_2_, H_3_ … H_p_) were recorded at the rate of 5.9 frames per second (t_0_, t_1_, t_2_, t_3_ … t_p_) without activating the vibration isolation platform. The fast Fourier transform was applied to each hologram to obtain their Fourier spectrum. The object wavefront terms corresponding to the two FOVs were filtered out from the Fourier spectrum and inverse Fourier transform to obtain the phase of each hologram separately (*ϕ*_0_, *ϕ*_1_, *ϕ*_2_, *ϕ*_3_ … *ϕ*_p_). Figure 6 depicts the scheme of the measurement of the temporal phase stability. The first hologram (H_0_) with a phase distribution *ϕ*_0_, was taken as a reference hologram for all the other holograms and the phase difference distributions were calculated by subtracting the phase of each hologram from the phase of the first hologram. It was assumed that the first reference hologram was undeformed and all the other holograms are deformed [43,44,45,46]. Each phase difference distribution’s 4000 pixels within the same area were selected to measure the standard deviation. The histograms of the standard deviation for two FOVs are shown in Figure 6b,c, respectively, demonstrating that the average mean fluctuation was 0.039 radians in both the FOVs, which is quite stable compared to a two-channel off-axis holographic setup [47]. The temporal stability of the proposed system was almost near to the Lloyd mirror interferometer-based digital holographic system (0.050 rad) as measured and depicted in Figure 6d.

### 3.4. Step Height Measurement Results by the Proposed System

Holographic interferometric methods have been demonstrated to be versatile tools for the solution of many NDT problems [48,49,50,51]. Due to the potential capability of the DH to retrieve the amplitude and phase information of the object, it can be used as a non-destructive, optical metrology and inspection tool in a wide range of applications. To prove the effectiveness of the proposed method, we experimentally demonstrated that the proposed system has the ability to be used to measure the step height of a resolution chart by extracting the phase information. In this experiment, two multiplexed digital holograms were recorded without and with the object in the test beam. A resolution chart (Model No.—R3L3S1N) and English letter (B) were used in this experiment.

**Figure 7:**
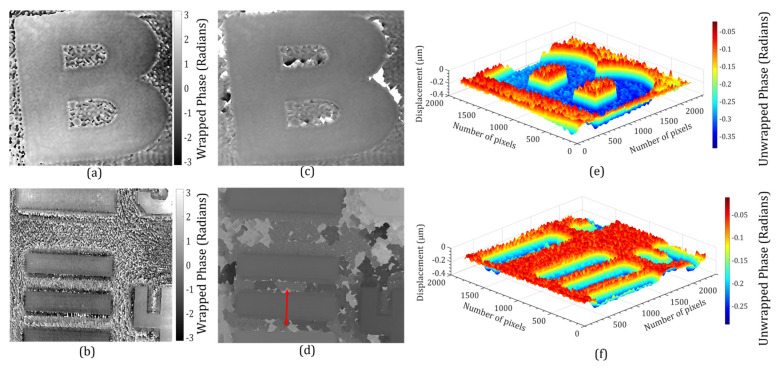
Experimental results of a step height multiplexed with the object (O_2_) and resolution chart: (**a**,**b**) wrapped phases for FOV1 and FOV2, (**c**,**d**) unwrapped phases, and (**e**,**f**) 3D height profile maps corresponding to (**c**,**d**).

**Figure 8:**
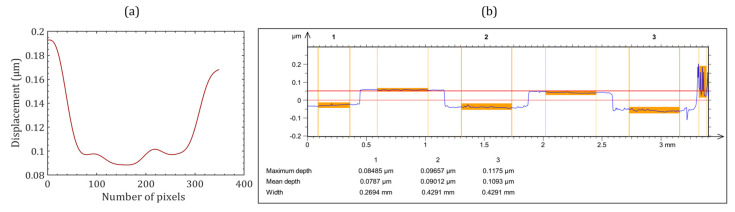
(**a**) Step height retrieved from the double side arrow line of the phase step region in Figure 7d and (**b**) Step height profile obtained by the commercial optical profiler.

The phase distributions of the object beams corresponding to the two FOVs were numerically reconstructed separately by the FDM from both digital holograms. Figure 7a,b show the phase difference between the two states of the object. The phase was calculated directly by modulo 2π subtraction. The numerically calculated phase remained wrapped in the range (−π, +π) radians. This 2π phase discontinuity was corrected by the Goldstein branch cut method [52] to obtain a continuous unwrapped phase, as shown in Figure 7c,d. The obtained continuous unwrapped phase distributions could further be used for measuring various physical parameters of the object under study, including deformation, displacement, height profile, stain or stress, vibration, refractive index, density, temperature, etc. We measured the height profiles of the objects corresponding to the two FOVs which were retrieved from the unwrapped phase (∆*ϕ*) profiles by using the relationship, $h=\frac{\lambda }{2\pi (\Delta n)}\Delta \phi$, where Δ*n* is the refractive index difference between object and air [53]. The 3D height profiles of the objects for both the FOVs are shown in Figure 7e,f. The plot of the measured height profile of the resolution chart across the vertical red arrow (Figure 7d) is depicted in Figure 8a.

The height profile obtained by averaging three measured values was 0.093 μm. The measured height was also validated with a commercial optical profilometer (CCI-Optics, Taylor Hobson Ltd., Leicester, UK); the measured plot is shown in Figure 8b. The measured height by the commercial optical profilometer was 0.096 μm (see Figure 8b), confirming that the proposed system provided consistent results. A difference of 4.1% was observed in the measurements using the proposed system relative to a commercial optical profilometer. The obtained results prove the effectiveness of the proposed off-axis DH system in optical metrology applications with a larger FOV. In the future, we will modify the proposed system for the non-destructive testing of industrial and engineering applications, where a large FOV is always desired. We will explore the system’s capability to measure the refractive index and temperature of gaseous flames. The existing digital holographic systems are restricted to image and/or measure these parameters for a few millimeters only. The system will be modified to extend the measurement range to a few centimeters. Further, the system will be modified to a compact microscope (digital holographic microscope) for biological imaging and investigations. In this system, we will measure the biophysical parameters of biological cells (human red blood cells) with the advantages of being compact, highly stable, double FOV, and a common-path system. 

## 4. Conclusions

In summary, we proposed and experimentally demonstrated a novel optical configuration of a common-path single-shot multiplexed off-axis dual FOV-DH. A highly stable, compact, and simple configuration of the off-axis DH was realized with Fresnel’s bi-mirror. The feasibility of the proposed setup was experimentally demonstrated by imaging and numerical reconstruction of dual FOVs. We also demonstrated the optical metrological application of the system by measuring the height profile of the object. The primary advantage of this method was its greater stability, and dual FOV as compared to the conventional holographic experimental setups. The proposed system is appropriate for the study of transparent/semi-transparent samples, i.e., phase objects. Therefore, the system could be used for prospective applications in microscopy, quantitative phase imaging, 3D imaging, and optical metrology. In the future, we aim to investigate new applications of the proposed system in digital holographic microscopy.

## Data Availability

Data underlying the results presented in this paper are not publicly available at this time but may be obtained by the corresponding author upon reasonable request.
